# AAV-glycine receptor α3 alleviates CFA-induced inflammatory pain by downregulating ERK phosphorylation and proinflammatory cytokine expression in SD rats

**DOI:** 10.1186/s10020-023-00606-9

**Published:** 2023-02-15

**Authors:** Hung-Chen Wang, Kuang-I Cheng, Kuang-Yi Tseng, Aij-Lie Kwan, Lin-Li Chang

**Affiliations:** 1 Department of Neurosurgery, Kaohsiung Chang Gung Memorial Hospital, Chang Gung University College of Medicine, Kaohsiung, Taiwan; 2 Department of Anesthesiology, Faculty of Medicine, College of Medicine, Kaohsiung Medical University, Kaohsiung, Taiwan; 3 Graduate Institute of Medicine, College of Medicine, Kaohsiung Medical University, Kaohsiung, Taiwan; 4 Department of Neurosurgery, Faculty of Medicine, College of Medicine, Kaohsiung Medical University, Kaohsiung, Taiwan; 5 Department of Microbiology and Immunology, Faculty of Medicine, College of Medicine, Kaohsiung Medical University, 100, Shih-Chuan 1st Road, Kaohsiung, 80708 Taiwan; 6 Graduate Institute of Clinical Medicine, College of Medicine, Kaohsiung Medical University, Kaohsiung, Taiwan; 7 Center for Infectious Disease and Cancer Research, Kaohsiung Medical University, Kaohsiung, Taiwan; 8 Department of Medical Research, Kaohsiung Medical University Hospital, Kaohsiung, Taiwan

**Keywords:** Adeno-associated virus, Extracellular signal-regulated kinase (ERK) phosphorylation, Glycine receptors, Prostaglandin E2, Inflammatory pain

## Abstract

**Background:**

Glycine receptors (GlyRs) play key roles in the processing of inflammatory pain. The use of adeno-associated virus (AAV) vectors for gene therapy in human clinical trials has shown promise, as AAV generally causes a very mild immune response and long-term gene transfer, and there have been no reports of disease. Therefore, we used AAV for GlyRα1/3 gene transfer in F11 neuron cells and into Sprague–Dawley (SD) rats to investigate the effects and roles of AAV-GlyRα1/3 on cell cytotoxicity and inflammatory response.

**Methods:**

In vitro experiments were performed using plasmid adeno-associated virus (pAAV)-GlyRα1/3-transfected F11 neurons to investigate the effects of pAAV-GlyRα1/3 on cell cytotoxicity and the prostaglandin E2 (PGE2)-mediated inflammatory response. In vivo experiment, the association between GlyRα3 and inflammatory pain was analyzed in normal rats after AAV-GlyRα3 intrathecal injection and after complete Freund's adjuvant (CFA) intraplantar administration. Intrathecal AAV-GlyRα3 delivery into SD rats was evaluated in terms of its potential for alleviating CFA-induced inflammatory pain.

**Results:**

The activation of mitogen-activated protein kinase (MAPK) inflammatory signaling and neuronal injury marker activating transcription factor 3 (ATF-3) were evaluated by western blotting and immunofluorescence; the level of cytokine expression was measured by ELISA. The results showed that pAAV/pAAV-GlyRα1/3 transfection into F11 cells did not significantly reduce cell viability or induce extracellular signal-regulated kinase (ERK) phosphorylation or ATF-3 activation. PGE2-induced ERK phosphorylation in F11 cells was repressed by the expression of pAAV-GlyRα3 and administration of an EP2 inhibitor, GlyRαs antagonist (strychnine), and a protein kinase C inhibitor. Additionally, intrathecal AAV-GlyRα3 administration to SD rats significantly decreased CFA-induced inflammatory pain and suppressed CFA-induced ERK phosphorylation, did not induce obvious histopathological injury but increased ATF-3 activation in dorsal root ganglion (DRGs).

**Conclusions:**

Antagonists of the prostaglandin EP2 receptor, PKC, and glycine receptor can inhibit PGE2-induced ERK phosphorylation. Intrathecal AAV-GlyRα3 administration to SD rats significantly decreased CFA-induced inflammatory pain and suppressed CFA-induced ERK phosphorylation, did not significantly induce gross histopathological injury but elicited ATF-3 activation. We suggest that PGE2-induced ERK phosphorylation can be modulated by GlyRα3, and AAV-GlyRα3 significantly downregulated CFA-induced cytokine activation.

**Supplementary Information:**

The online version contains supplementary material available at 10.1186/s10020-023-00606-9.

## Introduction

Pain is an obnoxious sensory and emotional experience due to an injury or disease of the somatosensory nervous system. It is known that ion channels and receptors in the dorsal root ganglia (DRG) and spinal cord are responsible for the detection of noxious stimuli, and their plasticity contributes to the increased severity of pain. Among these ion channel and receptors, they can be divided into activating receptors such as transient receptor protein vanilloid 1 (TRPV1) and *N*-methyl-d-aspartate (NMDA), and inhibitory receptors such as γ-Aminobutyric acid (GABA) and glycine receptor (GlyRs). Among them, TRPV1, NMDA and GABA receptors have been studied a lot (Naik et al. 2022; Aubrey and Supplisson 2018; Akhilesh et al. 2022a, b; Uniyal et al. 2022a). Inhibitory neurotransmission in the spinal cord is shaped by the balance between GABA receptor and GlyRs (Aubrey and Supplisson 2018). However, there are few studies about inflammatory proteins related to GlyRs, and the functional role for downstream proteins and signals mediated by GlyRs is unclear.

Glycine receptors (GlyRs) are anion-permeable pentameric ligand-gated ion channels that belong to the Cys-loop superfamily of ligand-gated ion channels (Lynch 2004). Previous studies found GlyRs in the brain, spinal cord, and DRG of mammals (Betz et al. 1991; Dutertre et al. 2012; Wang et al. 2018). GlyRs play critical roles in the mammalian central nervous system, including in motor coordination (Moraga-Cid et al. 2020). Hyperekplexia (Bode and Lynch 2014), epilepsy (Winkelmann et al. 2014), autism (Pilorge et al. 2016), and pain signaling (Zeilhofer 2005). There is also evidence showing that part of the spinal component of inflammatory hyperalgesia is the result of diminished glycinergic inhibition caused by the phosphorylation and inhibition of GlyRα3 (Harvey et al. 2004b; Ahmadi et al. 2002; Reinold et al. 2005; Hosl et al. 2006). Prostaglandin E2 (PGE2) is an important mediator of the pathogenesis of inflammatory diseases, mediated through activation of EP2 receptors and subsequent protein kinase A (PKA)-dependent phosphorylation of GlyRα3 (Moraga-Cid et al. 2020; Harvey et al. 2004b; Isensee et al. 2014). It has also been reported that protein kinase C epsilon (PKCε) plays a pivotal role in hyperalgesic priming (Reichling and Levine 2009). The PKA-mediated acute phase of hyperalgesia is evoked by PGE2, while PKCε mediates the prolonged phase (Dina et al. 2009; Khasar et al. 2008; Aley et al. 2000). PGE2 stimulates phosphorylated extracellular signal-regulated kinase (p-ERK) and interleukin (IL)-6 protein kinase pathways in DRG neurons (St-Jacques and Ma 2011). However, a previous study found that phosphorylated pan-PKC, p38, and c-Jun N-terminal kinase (JNK) were not altered following long-acting PGE2 analog treatment in DRG neurons (St-Jacques and Ma 2011). The downstream mechanism of GlyRs remains poorly understood. In our first in vitro experiment, we explored downstream proteins and signals mediated by GlyRs.

Adeno-associated virus (AAV) is a member of the parvovirus family that can be used to infect humans in clinical trials and in experimental animal models (Daya and Berns 2008). The use of AAV vectors for gene therapy in human clinical trials has shown promise, as AAV generally causes a very mild immune response and long-term gene transfer, and there have been no reports of disease. For tissue tropism, the existence of a variety of serotypes makes AAV gene therapy more attractive, since these vectors differ in infectivity rates and tissue specificity. Previous studies demonstrated extensive and effective transduction in the brain and spinal cord after AAV8 injection (Klein et al. 2008; Ayers et al. 2015). The F11 neuron line possesses many properties seen in nociceptive DRG neuronal cells. The transient transfection efficiency is approximately 50% for F11 neurons (Mahapatra et al. 2006; Jahnel et al. 2001) Compared with other cell lines, such as HEK293, F11 neuron lines are excellent proxies for the responses of true neurons. Therefore, we used AAV8 for GlyRα1/3 gene transfer in F11 neuron cells and investigated the effects and roles of plasmid adeno-associated virus (pAAV)-GlyRα1/3 on cell cytotoxicity and the PGE2-mediated inflammatory response. Furthermore, intrathecal AAV-GlyRα3 delivery into SD rats was evaluated in terms of its potential for alleviating CFA-induced inflammatory pain.

## Materials and methods

### Animal use and cell culture

F11 cells were purchased from the European Collection of Authenticated Cell Cultures (ECACC, 08062601) and cultured in DMEM supplemented with 10% FBS (both Invitrogen) in a humidified atmosphere at 37 °C with 5% CO_2_. The *Escherichia coli* DH5α strain was cultured at 37 °C in Luria–Bertani (LB) broth medium supplied with kanamycin (50 µg/ml) and was used as a host in transformation. The institutional review board of Kaohsiung Medical University, Kaohsiung, Taiwan approved the gene recombinant experiment in this study (KMU-106076 and 109033). We used 30 adult male SD rats weighing 250,300 g. The animals were housed in plastic cages with soft bedding with exposure to a 12-h light–dark cycle (light cycle: 07:00–19:00; dark cycle: 19:00–07:00) and access to food and water. All experimental procedures were approved by the Institutional Animal Care and Use Committee (approval no. 109148). All animal experiments in this study complied with the ARRIVE guidelines.

The current experiment selected AAV8 for GlyRα1/3 gene transfer. The experiments were divided into two parts. At the beginning of the in vitro experiment, we investigated the effects of pAAV-GlyRα1/3 on cell cytotoxicity and the PGE2-mediated inflammatory response in the F11 neuron line. In the second part of the in vivo SD rat experiment, we investigated intrathecal delivery of AAV-GlyRα3 to determine whether it may be a safe approach for downregulating CFA-induced inflammatory pain. CFA-injected rats were injected with CFA (100 μg/100 μl, *Mycobacterium tuberculosis*, Sigma, St. Louis, MO) was given through subcutaneous injections into the rat plantar of the left hind paw. The injection procedure was similar to previous studies by others (Weng et al. 2015; Uniyal et al. 2022b). Furthermore, intrathecal AAV-GlyRα3 delivery into SD rats was evaluated in terms of its potential for alleviating CFA-induced inflammatory pain. Rats were divided into four groups: an intrathecal AAV-GlyRα3 plus CFA injection group (Gα3F group, n = 9); an intrathecal AAV plus CFA injection group (GVF group, n = 6); an intrathecal NaCl plus CFA injection group (GNF group, n = 9); and a normal control group (N group, n = 6).

### pAAV-GlyRα1 and pAAV-GlyRα3 recombinant vector construction

In this study, the production of AAV8 carrying GlyRα1 or GlyRα3 fragment from the brains of SD rats was described. First, total RNA was extracted from the brains of SD rats and then reverse transcribed to cDNA using an MMLV reverse transcription kit (Protech). GlyRα1 and GlyRα3 fragments were PCR-amplified from rat cDNA using the following primers. GlyRα1 (forward: 5′-ACAGCGGCCGCACCATGTACAGCTTCAACACTCTG-3′, reverse: 5′-GGCGATATCTCACTTGTTGTGGACGTC-3′); GlyRα3 (forward: 5′-ACAGCGGCCGCACCATGCCTTGGATAAGACTG-3′, reverse: 5′-GGCGATATCTTAATCTTGCTGATGATGAATG-3′). These two GlyRα fragments of PCR products were eluted and purified from low-melting-point agarose gels (Thermo Fisher Scientific) and then ligated into the pCR™-Blunt II-TOPO® vector by Zero Blunt® TOPO® PCR cloning kits (Invitrogen) to construct the recombinant vectors pBlunt-GlyRα1 and pBlunt-GlyRα3. Later, these two recombinant vectors were transferred to *Escherichia coli* DH5α competent cells by transformation. Extraction and purification of recombinant vectors pBlunt-GlyRα1 and pBlunt-GlyRα3 from *Escherichia coli* DH5α cells was carried out with a Presto™ Mini plasmid kit (PDH100, PDH300) (Geneaid, Taiwan). The accuracy of the GlyRα1 and GlyRα3 fragments ligated with the pCR™Blunt II-TOPO® vector was confirmed by PCR (primers for GlyRα1 forward: 5′-AAGAATTTCCCGATGGACGTA-3′, reverse: 5′-GTAGTGCTTGGTGCAGTA-3′; primers for GlyRα3 (forward: 5′-AAACACTACAATACAGGAAAGTTTAC-3′, reverse: 5′-CAGTGGTGATACCCAACG-3′) and DNA sequencing. Second, NotI and EcoRV restriction enzymes were selected to cleave pBlunt-GlyRα1, pBlunt-GlyRα3 recombinant DNA and the pAAV-IRES-GFP expression vector. The restriction products were detected in low-melting-point agarose gels electrophoresed, eluted, purified and subjected to ligation reaction by T4 DNA ligase (BioLabs) to construct recombinant vectors pAAV-GlyRα1 (Additional file 1: Fig. S1A) and pAAV-GlyRα3 (Additional file 1: Fig. S1B). Last, by transformation, these two recombinant vectors were then transferred into *Escherichia coli* DH5α competent cells. Recombinant vectors pAAV-GlyRα1 and pAAV-GlyRα3 from *Escherichia coli* DH5α cells were extracted and purified again with a Presto™ Mini plasmid kit (PDH100, PDH300) (Geneaid, Taiwan). The accuracy of the GlyRα1 and GlyRα3 fragments from pAAV-GlyRα1 and pAAV-GlyRα3 was confirmed by PCR (the primers for GlyRα1 were forward: 5′-AAGAATTTCCCGATGGACGTA-3′, reverse: 5′-GTAGTGCTTGGTGCAGTA-3′; the primers for GlyRα3 were forward: 5′-AAACACTACAATACAGGAAAGTTTAC-3′, reverse: 5′-CAGTGGTGATACCCAACG-3′) and DNA sequencing. The correct recombinant clones containing pAAV-GlyRα1 or pAAV-GlyRα3 were stored at − 80 °C until further use.

### pAAV-GlyRα1, pAAV-GlyRα3 transfection into F11 cells

F11 cells were seeded into a 6-well plate at a density of 3 × 10^5^ cells/well. When cell confluence reached 70%, the cells were transfected with pAAV-GlyRα1 or pAAV-GlyRα3 (2 μg or 5 μg) recombinant vectors with Lipofectamine® 2000 transfection reagent (Invitrogen) at 37 °C with 5% CO_2_ for 24, 48, and 72 h. Transfection efficiency was assayed by assessing the green fluorescent protein (GFP) emitted from the pAAV-GlyRα1 or pAAV-GlyRα3 recombinant vectors inside F11 cells.

### Cell viability assay

The effect of pAAV, pAAV-GlyRα1 or pAAV-GlyRα3 as well as PGE2 on F11 cells was determined by MTT assay. F11 cells (7 × 10^4^ cells/well) were seeded in a 24-well plate, and after 24 h in culture, 2 μg of pAAV, pAAV-GlyRα1, pAAV-GlyRα3 or Lipofectamine alone was used to transfect F11 cells for 48 h. In addition, F11 cells were cultured for 48 h, serum-free medium replaced the initial medium and was cultured for another 24 h, and then, PGE2 (100 μM) was added for 60 min. Finally, cell viability was assessed by MTT assay kit (Abcam) according to the manufacturer’s protocol. Briefly, culture medium was removed, MTT reagent was added to each well, and the cells were incubated for 2–6 h at 37 °C. The MTT reagent was removed, DMSO was added, and the cells were incubated for 5 min. The supernatant was collected, and the absorbance was measured at OD 550–600 nm. F11 cells that were neither transfected nor subjected to Lipofectamine-only treatment were used as controls.

### Virus production and titration

Virus preparation was performed at the AAV Core, Institute of Biomedical Sciences, Academia Sinica, Taipei, Taiwan. In brief, the pAAV or pAAV-GlyRα3 plasmid, pHelper plasmid, and pAAV-RC plasmid were cotransfected into human embryonic kidney 293 cells (cat. no. CRL-1573; ATCC, Manassas, VA, USA). Virus was purified using a CsCl gradient, titers were measured as virus genomes (vg) per milliliter, and eGFP levels were detected using Roche light cycler® real-time PCR.

### Intrathecal injection of NaCl, AAV, or AAV-GlyRα3 and intraplantar CFA administration

According to our previous work described by Wang et al. (2016, 2018), we placed rats in the prone position and made a 2-cm longitudinal skin incision on the midline immediately above the L5 and L6 spinal process. The L5/L6 interspinous ligaments were incised, and one-half of the anterior L6 spinal process was removed, which allowed direct visualization of the L5/6 ligamentum flavum. Under a surgical microscope (Leica M690; Leica, Wetzlar, Germany), NaCl, AAV viral particles (2.5 × 10^12^ vg), or AAV-GlyRα3 viral particles (2.5 × 10^12^ vg) were intrathecally injected into the subarachnoid space of the cauda equina using a 30-gauge needle (0.3 mm × 1.3 mm; BD, Franklin Lakes, NJ, USA). Five minutes after injection, the needle was withdrawn, and using a microscope, we checked that no fluid was leaking. The wound was approximated with surgical sutures, and then, the animals were placed in a recovery cage to recover, and they were monitored until they resumed normal activity. CFA (100 μg/100 μl) was injected through the intraplantar eight weeks later.

### Behavioral test

According to our previous report, Dynamic Plantar Aesthesiometer (UgoBasil, Monvalle VA, Italy) with Von Frey filaments test machine was used for automated mechanical stimulation and allodynia measurement (Wang et al. 2016; Chang et al. 2020). The latency of foot withdrawal from a noxious heat stimulus was measured by a device consisting of a light box with a glass plate on top (Model 7370 Plantar Test, Ugo Basile, Varese, Italy) (Wang et al. 2016, 2018; Chang et al. 2020). Behavior was assessed at day 0 (baseline); weekly for two months after intrathecal injection of AAV-GlyRα3, AAV, or NaCl; and daily for four days after CFA injection. The rats were then anesthetized under 2–3.5% isoflurane/O_2_ and sacrificed; L5 DRGs were collected.

### Western blotting

To determine the effect of PGE2, recombinant pAAV-GlyRα1 or pAAV-GlyRα3 on inducing ERK phosphorylation and ATF-3 activation, 2 μg pAAV, pAAV-GlyRα1 or pAAV-GlyRα3 was selected to transfect F11 cells (3 × 10^5^ cells/well), which were seeded into 6-well plates, and incubated for 48 h. In addition, pAAV, pAAV-GlyRα1 or pAAV-GlyRα3 transfected F11 cells were cultured for 48 h, serum-free medium replaced the initial medium and was cultured for another 24 h, and then, PGE2 (100 μM) was added for 60 min. F11 cells treated with PGE2 for 5, 15, 30 and 60 min alone were also included in the present study. To investigate the pathway of PGE2-induced ERK phosphorylation, the glycine receptor antagonist strychnine and the EP2 receptor antagonist PF-04418948 were added and incubated for 24 h. One hour after PGE2 administration, cell pellets were harvested, and ERK phosphorylation was measured. The protein kinase C (PKC) inhibitor G06983 (3 μM) was applied 30 min before PGE2 treatment. One hour after PGE2 application, the cells were harvested for protein extraction.

For western blotting, cells seeded as described above and L5 DRGs from SD rats were harvested and homogenized in RIPA lysis buffer (50 mM Tris pH 7.4, 150 mM NaCl, 1 mM EDTA, 0.1% SDS, 1% NP-40, and 0.5% sodium deoxycholate) containing protease inhibitor cocktail (Roche, Germany). The protein concentration was determined using a Bio-Rad protein assay kit (Bio-Rad, Hercules, CA, USA). Twenty micrograms of total protein was loaded into 8% (w/v) sodium dodecyl sulfate-polyacrylamide gels and then transferred to polyvinylidene fluoride membranes (Millipore, Bedford, MA, USA). The filters were incubated with rabbit monoclonal anti-phospho-p44/42 MAPK (Erk1/2) (Cell Signaling Technology, 4370p), rabbit monoclonal anti-p44/42 MAPK (Erk1/2) (Cell Signaling Technology, 4695p), rabbit monoclonal anti-phospho-p38 MAPK (mitogen-activated protein kinase; Cell Signaling Technology, Boston, MA, USA), rabbit monoclonal anti-p38 MAPK (Cell Signaling Technology), rabbit anti-ATF-3 (NOVUS, NBP1-85816, USA), or mouse monoclonal anti-actin (MAB1501; Indianapolis, IN, USA) primary antibodies. This treatment was followed by reaction with horseradish peroxidase-conjugated goat anti-mouse IgG or goat anti-rabbit IgG secondary antibody (Santa Cruz Biotechnology). We use Western Blot Stripping Buffer (Biomate, BS-20-500, Taipei, Taiwan) to remove antibodies from blots membrane in case of re-probing is needed. The intensity of each band was visualized with ECL western blotting detection reagents (Amersham Biosciences, Tokyo, Japan). Protein expression was measured and normalized to the density of the corresponding internal β-actin control. Then, the expression levels of the experimental groups relative to the control group were calculated.

### Immunofluorescence

The dissected L5 DRG tissues were then fixed in 4% (w/v) paraformaldehyde in a 0.1 mol/l phosphate buffer (pH 7.4) and saturated in 10–30% (w/v) sucrose in 0.02 mol/l PBS (pH 7.4). After embedding the tissues in optimal cutting temperature (OCT) compound, the morphological integrity of the DRG sections was determined by hematoxylin eosin (HE) staining, and then, L5 DRGs (12 µm) were prepared for immunostaining. Tissue sections were incubated with the following primary antibodies: rabbit anti-phospho-p44/42 MAPK (Erk1/2) monoclonal antibody (1:200, Cell Signaling, #4370), rabbit anti-phospho-p38 monoclonal antibody (1:200, Cell Signaling, #9215), mouse anti-NeuN monoclonal antibody (1:200, Merck, MAB377), mouse anti-neurofilament 200 monoclonal antibody (1:200, Merck, MAB5262), rabbit anti-glial fibrillary acidic protein (GFAP) polyclonal antibody (1:500, Merck, AB5804), and rabbit anti-ATF3 polyclonal antibody (1:200, Novus, NBP1-85816). These incubations were followed by incubation with secondary antibodies: Cy3-conjugated goat anti-mouse IgG antibody (1:200, Jackson ImmunoResearch, Code 115-165-003), Cy3-conjugated goat anti-rabbit IgG antibody (1:200, Jackson ImmunoResearch, Code 111-165-003), or DyLight® 405 goat anti-mouse IgG (1:200, Thermo Fischer, 35501BID). The immunoreactivity (IR) of each section was examined. Images were captured using a Leica DMi8 microsystem or a Zeiss LSM 700 confocal microscope (Zeiss, Jena, Germany).

### Measurement of cytokines by ELISAs

To investigate the possible role of glycine receptors in modulating CFA-induced cell inflammatory reactions, according to the transfection efficiency assay described above, 2 μg pAAV, pAAV-GlyRα1 or pAAV-GlyRα3 was selected to transfect F11 cells (3 × 10^5^ cells/well), which were seeded into 6-well plates, for 48 h. Serum-free medium replaced the initial medium and was incubated for another 24 h, and then, the cells were treated with CFA (100 ng) for another 6 h. Supernatants were collected for analyses of cytokines, including IL-1β, TNF-α, and IL-6, by ELISA (R&D Inc., Minneapolis, MN, USA). F11 cells without pAAV, pAAV-GlyRα1 or pAAV-GlyRα3 transfection or CFA treatment were used as controls. In an in vivo study, to investigate whether intrathecal delivery of AAV-GlyRα3 alleviates CFA-induced inflammatory response, L5 DRGs (25 μg/100 μl) were collected for cytokine analyses.

### Statistical analysis

The results are presented as the mean ± SE. Analytical statistics were performed using the SPSS (version 20) software package. Western blots were determined by one-way ANOVA analysis followed by the least significant difference test for multiple post hoc analyses. Behavioral responses were assessed using the Mann–Whitney U test. Differences were considered statistically significant at *p < 0.05, **p < 0.01, ***p < 0.001. Other statistically significant at ^#^*p* < 0.05, ^##^*p* < 0.01, ^###^*p* < 0.001 were used in behavioral responses.

## Results

### pAAV/pAAV-GlyRα1/3 transfection does not induce cells cytotoxicity

The time schedule for measuring the transfection efficiency of pAAV-GlyR α1/pAAV-GlyR α3 is shown in Additional file 2: Fig. S2A. Though different pAAV-GlyRα1/3 transfection dosage (2 μg or 5 μg) lead to similar transfection efficiency was observed. Increased transfection efficiency was found after long-duration pAAV-GlyRα1 (Additional file 2: Fig. S2B) or pAAV-GlyRα3 transfection (Additional file 2: Fig. S2C). Therefore, transfection with 2 μg pAAV, pAAV-GlyRα1, or pAAV-GlyRα3 for 48 h was used in our study, including for the MTT assay. Additional file 2: Fig. S2D shows the time schedule for measuring cell viability by MTT assay. The viability of F11 cells transfected using pAAV, pAAV-GlyRα1, pAAV-GlyRα3, or Lipofectamine alone, as well as those treated with PGE2, was evaluated. Compared with the control, cell viability was significantly decreased in cells transfected with pAAV, pAAV-GlyRα1, or pAAV-GlyRα3, as well as those treated only with Lipofectamine. Cell viability was lower (but not significantly) in the pAAV, pAAV-GlyRα1 or pAAV-GlyRα3 groups than in the Lipofectamine-treated group (Additional file 2: Fig. S2E). This finding indicates that decreased cell viability in the pAAV-, pAAV-GlyRα1- or pAAV-GlyRα3-transfected groups was caused by the Lipofectamine transfection reagent. However, cytotoxicity caused by pAAV, pAAV-GlyRα1 or pAAV-GlyRα3 transfection cannot be ruled out completely. Furthermore, cell toxicity caused by PGE2 treatment was not found in this study (Additional file 2: Fig. S2E).

### pAAV/pAAV-GlyRα1/3 transfection does not induce ERK phosphorylation or ATF-3 activation

#### pAAV-GlyRα3 suppresses PGE2-induced ERK phosphorylation

**Fig. 1:**
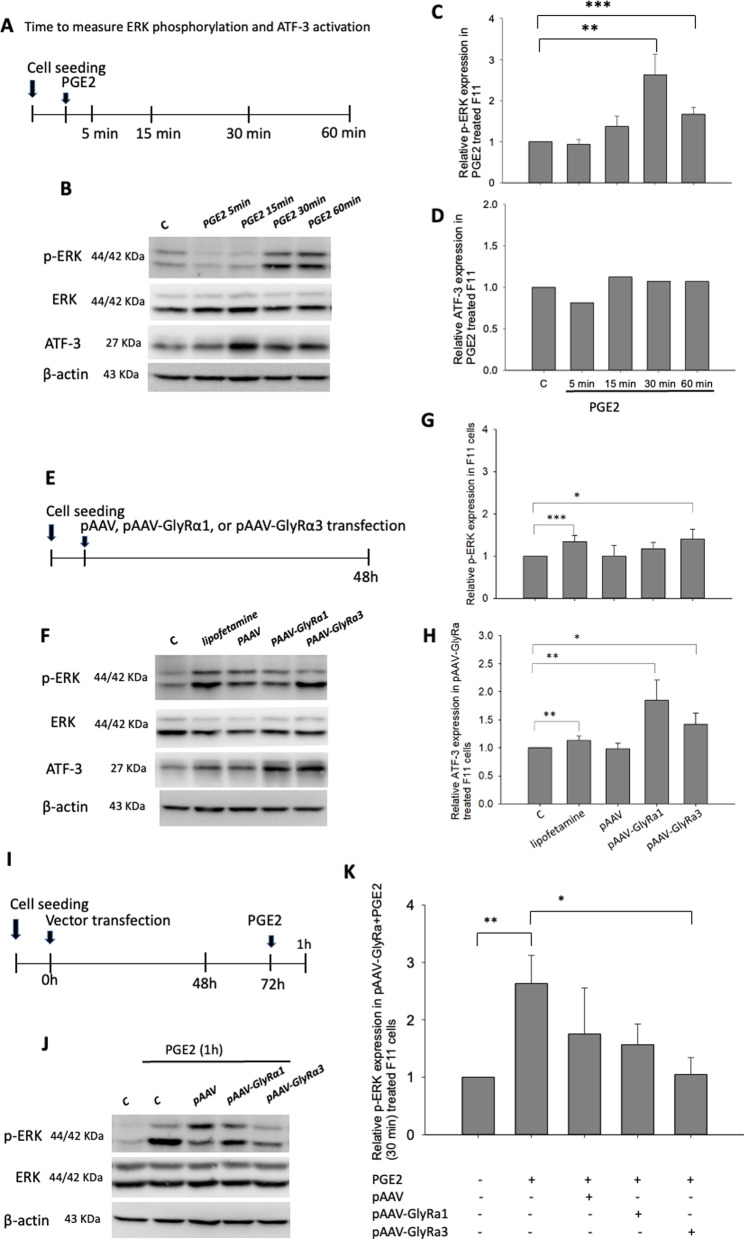
Effect of PGE2 and pAAV/pAAV-GlyR1/3 on ERK phosphorylation and ATF-3 activation in F11 cells. **A** Time schedule of PGE2 (100 µM) administration and the time when cells were collected to measure ERK phosphorylation and ATF-3 activation. F11 cells were seeded into 6-well plates, and 24 h later, PGE2 was applied for 5, 15, 30 or 60 min. Finally, these treated cells were harvested for protein extraction. **B** Western blot images and quantitative evaluation of **C** p-ERK and **D** ATF-3 activation in PGE2-treated F11 cells. **E** Time schedule of F11 cells transfected with 2 μg pAAV, pAAV-GlyRα1 or pAAV-GlyRα3 for 48 h. Then, cell pellets were harvested for protein extraction. **F** Images of western blots and quantitative evaluation of **G** p-ERK and **H** ATF3 expression in pAAV-, pAAV-GlyR1 or pAAV-GlyRα3-transfected F11 cells are shown. **I** Time schedule to evaluate the effect of pAAV, pAAV-GlyRα1/3 transfection on PGE2 (100 µM)-induced ERK phosphorylation in F11 cells. F11 cells were transfected with 2 μg pAAV, pAAV-GlyRα1 or pAAV-GlyRα3 for 48 h. Serum-free medium replaced the initial medium and was incubated for another 24 h, and the cells were then treated with PGE2 for 60 min. The cells were harvested for **J** western blotting of phosphorylated ERK, and **K** quantification of phosphorylated ERK in F11 cells is shown. F11 cells without vector transfection or PGE2 treatment were used as controls. All the data are expressed as the fold change measured in from three to five independent experiments. *p < 0.05, **p < 0.01, and ***p < 0.001, one-way ANOVA

The time schedule of PGE2 administration and cell collection for measuring ERK phosphorylation and ATF-3 is shown in Fig. 1A. The western blotting results indicated that phosphorylation of ERK increased significantly 30–60 min after administration of PGE2 (100 ng) (Fig. 1B, C). Importantly, PGE2 did not correlate with neuronal cell injury or ATF-3 activation (Fig. 1B, D) or p38 phosphorylation (Additional file 3: Fig. S3).

The time schedule of 48-h pAAV or pAAV-GlyRα1/3 transfection and cell collection for measuring ERK phosphorylation and ATF-3 activation is shown in Fig. 1E. We found that Lipofectamine induced significant ERK phosphorylation (Fig. 1F, G) and ATF-3 activation (Fig. 1F, H), although pAAV-GlyRα3 transfection was superior for inducing ERK phosphorylation (Fig. 1F, G) and ATF-3 activation (Fig. 1F, H) compared to that of the normal control. However, there was no significant difference between the Lipofectamine-treated group and pAAV-GlyRα1/3 transfection groups. This finding indicates that neither pAAV-GlyRα1 nor pAAV-GlyRα3 transfection causes ERK phosphorylation or ATF-3 activation. Furthermore, time schedule to examine the effect of vectors, including pAAV-GlyRα1 and pAAV-GlyRα3, on PGE2-induced ERK phosphorylation in F11 cells is shown in Fig. 1I. We found that PGE2 administration induced ERK phosphorylation; however, pretransfected pAAV-GlyRα3 (but not pretransfected pAAV or pAAV-GlyRα1) significantly suppressed PGE2-induced ERK phosphorylation (Fig. 1J, K).

### Blocking the glycine receptor inhibits PGE2-induced ERK phosphorylation

**Fig. 2:**
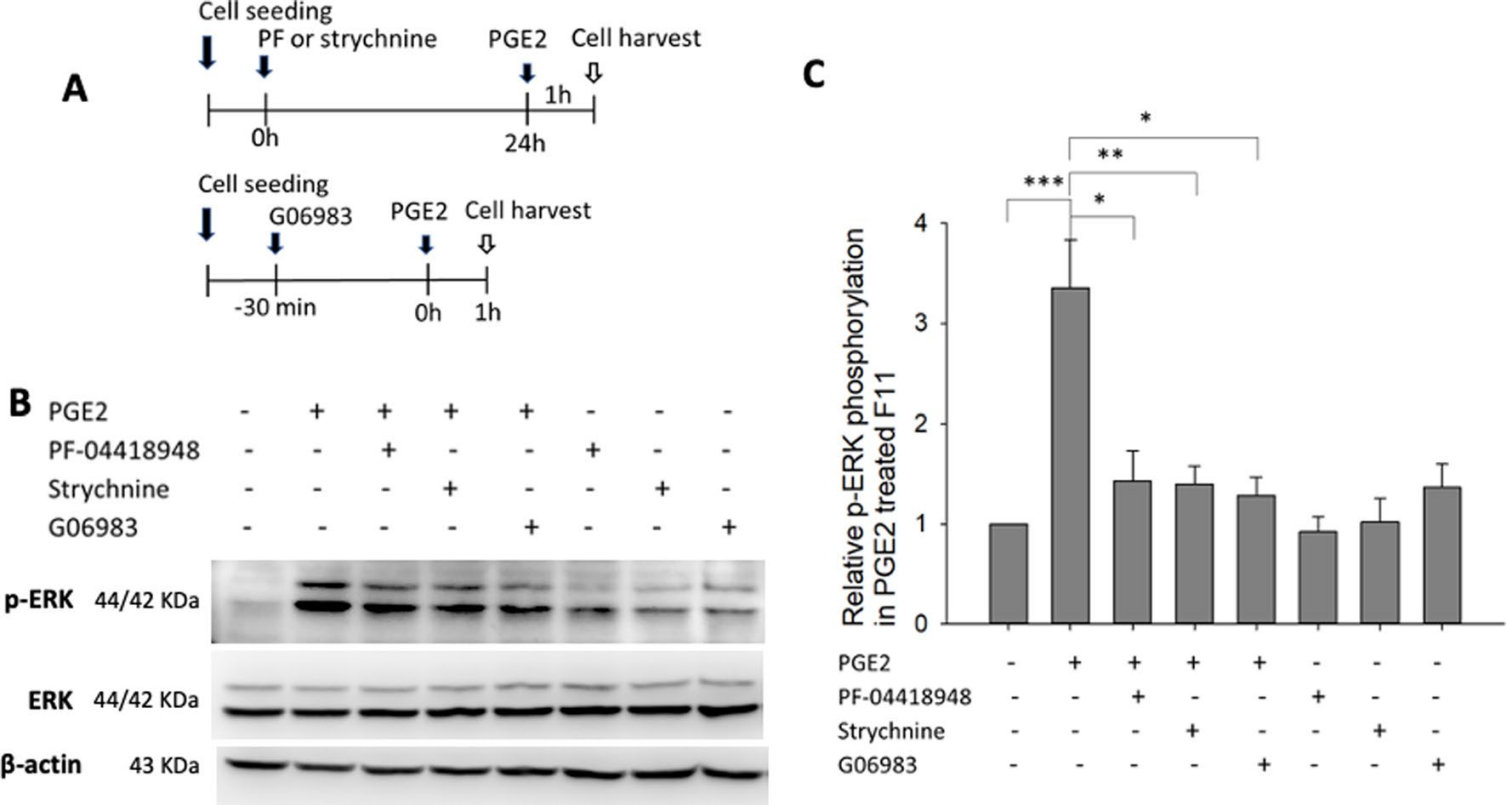
Effects of prostaglandin EP2-receptor antagonist (PF-04418948), glycine-receptor antagonist (strychnine), and PKC inhibitor (G06983) on PGE2-induced ERK phosphorylation. **A** Time schedule in which F11 cells were seeded into a 6-well plate for 24 h; PF-04418948 (10 μM) or strychnine (10 μM) was applied and incubated for 24 h before PGE2 treatment. G06983 (3 μM) was applied 30 min before PGE2 treatment. One hour after PGE2 (100 μM) application, cells were harvested for protein extraction. **B** Western blotting of ERK phosphorylation and **C** quantification of ERK phosphorylation in F11 cells. F11 cells without inhibitors or PGE2 treatment were used as a control. The data are presented on the basis of from three to five independent experiments. *p < 0.05, **p < 0.01, and ***p < 0.001, one-way ANOVA

Strychnine is an inhibitor of postsynaptic GlyRs. We examined whether EP2 or glycine receptors were critical for PGE2‐induced ERK phosphorylation in F11 cells. Figure 2A shows the time schedule for the application of the EP2- and glycine-receptor antagonists PF‐04418948 and strychnine. The results showed that PF‐04418948 and strychnine downregulate PGE2-induced ERK phosphorylation, suggesting that EP2 and GlyRs are essential to PGE2-induced ERK phosphorylation (Fig. 2B, C). In addition, the broad-spectrum PKC inhibitor G06983 administered 30 min before PGE2 treatment also led to a significant reduction in PGE2-induced ERK phosphorylation (Fig. 2B, C).

### Neuron cytotoxicity and ATF-3 expressions in DRG

**Fig. 3:**
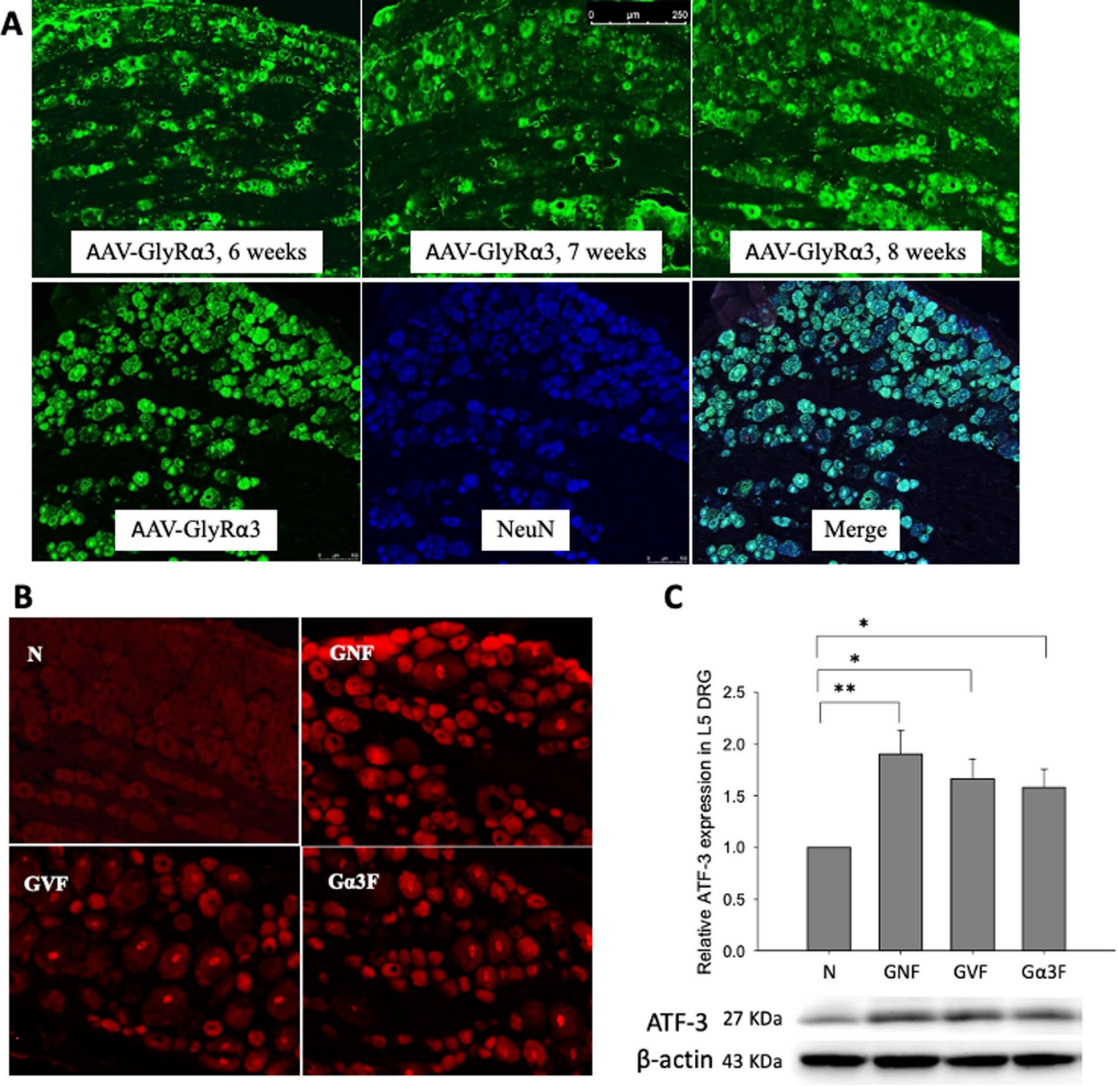
Immunofluorescence images of AAV-GlyRα3, and ATF-3 expression in the DRG. **A** Expression of green fluorescent protein (GFP) in L5 DRGs was detected by immunofluorescence six to eight weeks after intrathecal AAV-GlyRα3 (2.5 × 10^12^ vg) injection. Double immunofluorescence staining showing GlyRα3 and NeuN co-localization in the L5 DRG. Positive for GlyRα3 are shown in green, positive for NeuN are shown in blue, merge images of GlyRα3 and neuron are indicated in peacock blue. A injury assessment by ATF-3 expression of DRG tissue sections from CFA (100 μl) subcutaneous hind paw injection. Group intrathecal NaCl plus CFA injection (GNF), group intrathecal AAV (2.5 × 10^12^ vg) plus CFA injection (GVF), and group intrathecal AAV-GlyRα3 (2.5 × 10^12^ vg) plus CFA injection (Gα3F) are compared. ATF-3 expression in the DRG was measured by **B** immunofluorescence and **C** western blotting. The data represent the means ± SE (n = 6–9 per group). One-way ANOVA. *p < 0.05, **p < 0.01, ***p < 0.001. ATF-3 indicates activating transcription factor; scale bars: 250 μm

In vivo SD rat experiment, we investigated whether intrathecal delivery of AAV-GlyRα3 is safe for downregulating CFA-induced inflammatory pain. Firstly, AAV-GlyRα3 expression in the L5 DRG neuron was assessed by immunofluorescence to verify successfully delivering AAV-GlyRα3 into peripheral nerve after intrathecal AAV-GlyRα3 injection in SD rats. Figure 3A shows that the expression of green fluorescent protein (GFP) was observed six weeks after intrathecal AAV-GlyRα3 injection into SD rats and was magnified in L5 DRGs at eight weeks. Double immunofluorescence staining showing GlyRα3 and NeuN co-localization in the neuron of L5 DRG (Fig. 3A). Therefore, for this experiment, CFA intraplantar injection was administered 8 weeks after the intrathecal injection of NaCl, AAV, or AAV-GlyRα3. Furthermore, intrathecal NaCl plus CFA injection (GNF group), AAV plus CFA injection (GVF group), and AAV-GlyRα3 plus CFA injection (Gα3F group) significantly upregulated ATF-3 expression in the L5 DRGs in comparison with the normal control (Fig. 3B, C). Although there was no significant difference, slightly decreased ATF-3 expression in the Gα3F group was found when the Gα3F and GNF groups were compared in the study. The histopathological injury to DRG sections was determined by HE staining (data not shown). In terms of the results of HE histopathologic staining and ATF-3 expression in L5 DRG, we suggest that following AAV, AAV-GlyRα3 intrathecal administration, DRG neurons maintained their integrity; however, slight neuron injury can be induced following intrathecal injection (Fig. 3B, C).

### Intrathecal AAV-GlyRα3 administration significantly decreased CFA-induced inflammatory pain

**Fig. 4:**
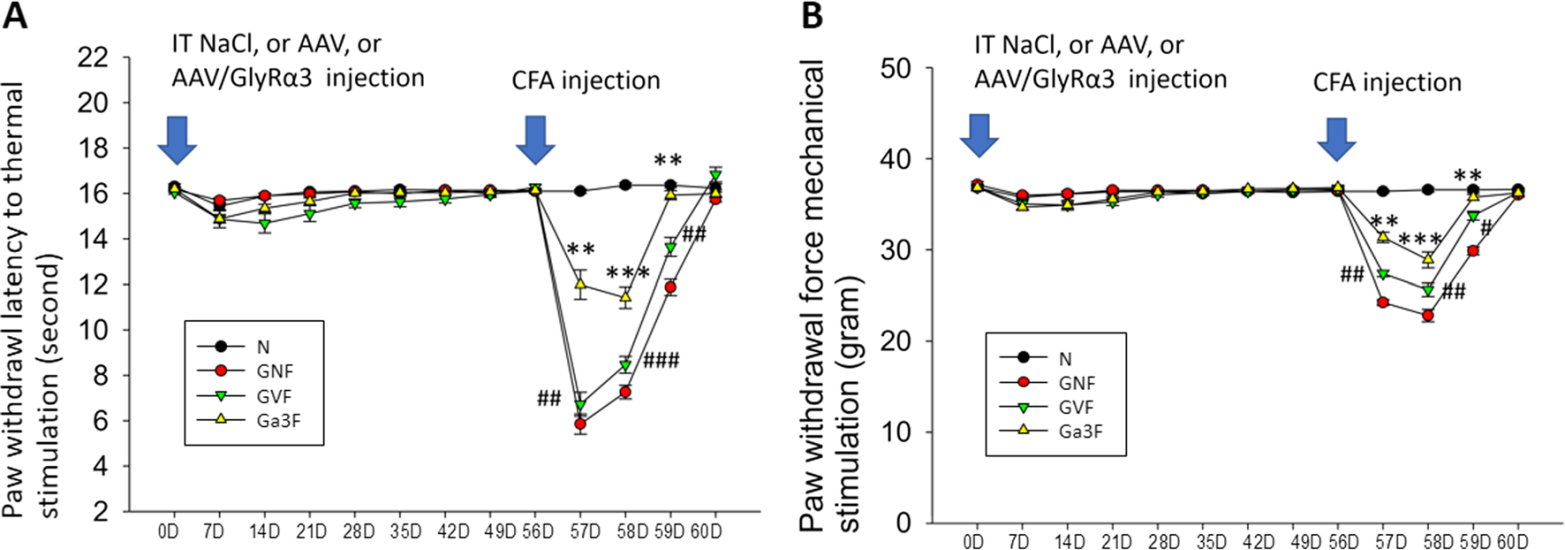
Behavioral responses to thermal and mechanical stimuli on hind paws. The hind paw withdrawal response to **A** thermal hyperalgesia and **B** mechanical allodynia was assessed at day 0 (baseline); weekly for two months after intrathecal injection of AAV-GlyRα3 (2.5 × 10^12^ vg), AAV (2.5 × 10^12^ vg), or NaCl; and daily for four days after CFA (100 μl) injection. Mann–Whitney U test, **p < 0.01, ***p < 0.001 (used in comparison between GNF and Gα3F groups). ^#^*p* < 0.05, ^##^*p* < 0.01, ^###^*p* < 0.001 (used in comparison between GVF and Gα3F groups); D, days

The inflammatory response induced by CFA injection is known to be a critical trigger of the pathological changes that produce inflammatory pain. Among the intrathecal NaCl, AAV, and AAV-GlyRα3 injection groups, the hind paw withdrawal response to noxious heat stimulus (thermal hyperalgesia, Fig. 4A) and mechanical stimulus (mechanical allodynia, Fig. 4B) was not significantly different from baseline up to two months before CFA hind paw injection. This observation indicates that inflammatory pain is not caused by intrathecal injection. However, inflammatory pain was induced shortly thereafter by subcutaneous plantar CFA injection and lasted for two days in intrathecal AAV-GlyRα3 injection group (Gα3F group, Additional file 4: Fig. S4A) and lasted for three days in intrathecal AAV, NaCl injection groups (GVF, GNF groups, Additional file 4: Fig. S4B, C) then back to normal response in the fourth day. But pain was significantly alleviated in intrathecal AAV-GlyRα3 administration group (Gα3F group) and the effect can last for three days no matter thermal or mechanical stimulation as compare with GNF group (Fig. 4, indicated by symbol *). Though mild pain alleviating effect was found after intrathecal AAV injection in the current study. However, significant difference of pain mitigating effect was observed when compared intrathecal AAV-GlyRα3 group with intrathecal AAV group (Fig. 4, indicated by symbol #). These results showed a significant analgesic effect was due to intrathecal AAV-GlyRα3 administration in a CFA-induced rat model.

### AAV-GlyRα3 suppresses CFA-induced ERK phosphorylation in L5 DRGs

**Fig. 5:**
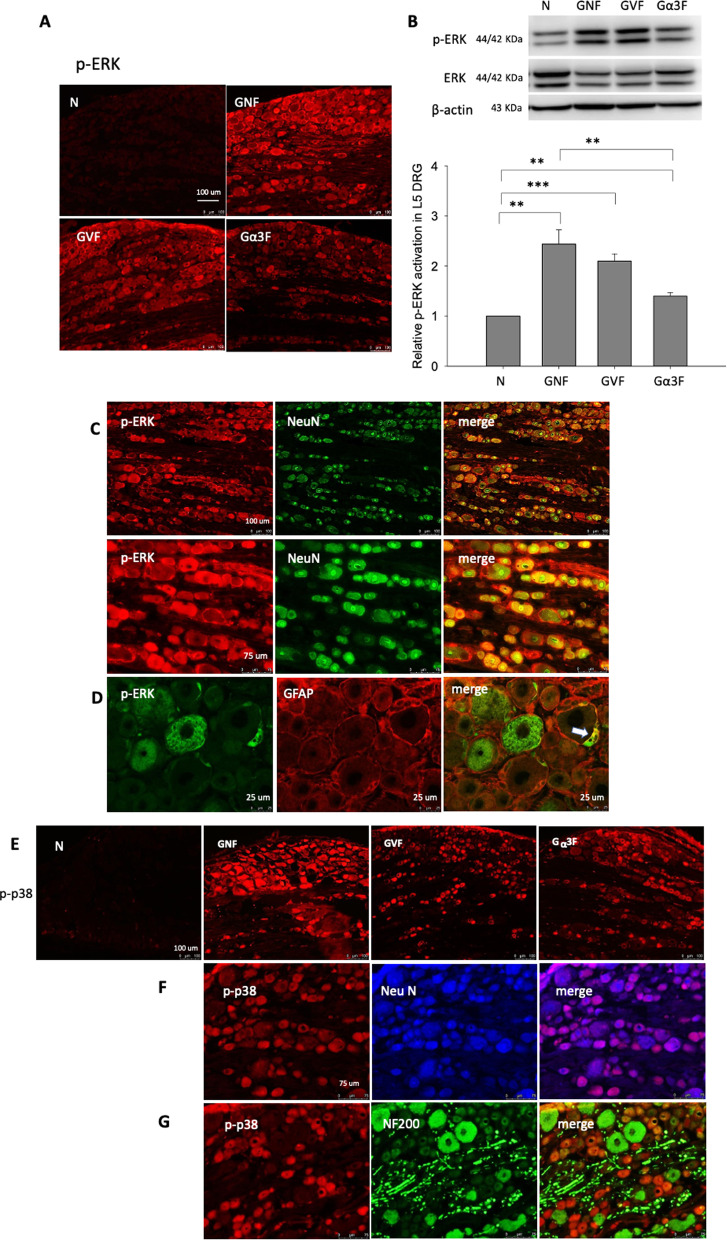
Effects of AAV-GlyRα3 on ERK and p38 phosphorylation in CFA‑treated rats. Intrathecal NaCl, AAV (2.5 × 10^12^ vg), or AAV-GlyRα3 (2.5 × 10^12^ vg) injection 8 weeks later CFA (100 μg/100 μl) was given through hind paw injection. After 2 days of CFA treatment, L5 DRGs were collected to evaluate ERK and p38 phosphorylation. By **A** immunofluorescence, **B** western blotting and quantification, a magnification image showing the induction of ERK phosphorylation in the GNF and GVF groups. CFA induces sustained activation of ERK, which was repressed in the Gα3F group. Double immunofluorescence labeling of **C** p-ERK with NeuN and **D** p-ERK with GFAP indicated colocalization of p-ERK with NeuN in neurons and satellite glial cell in the DRG. **E** Immunofluorescence imaging revealed p38 phosphorylation in the GNF, GVF and Gα3F groups. Double immunofluorescence labeling of **F** p-p38 with NeuN and **G** p-p38 with NF200 revealed p-p38 expression in small neurons. The data represent the means ± SE (n = 6–9 per group). One-way ANOVA. *p < 0.05, **p < 0.01, ***p < 0.001. Scale bars: 50–100 μm

Immunofluorescence and western blotting were used to detect ERK and p38 signaling pathway activation. In comparison with normal control group, GNF, GVF, and Gα3F groups significantly induced ERK activation in L5 DRG (Fig. 5A, B). However, the current in vivo results showed that AAV-GlyRα3 can significantly repress ERK phosphorylation in DRG when compared GNF group with Gα3F groups (Fig. 5A, B). Double immunofluorescent labeling of p-ERK with NeuN (Fig. 5C) or GFAP (Fig. 5D) revealed that p-ERK could be located in neurons (Fig. 5C) and satellite glial cells (Fig. 5D) in the DRG. However, CFA-induced p38 phosphorylation was not suppressed by AAV-GlyRα3 intrathecal administration (Fig. 5E). Double immunofluorescent labeling with p-p38 and NeuN or p-p38 and NF200 showed that p-p38 was expressed on small neurons (Fig. 5F, G).

### AAV-GlyRα3 suppresses cytokine activation in the DRG of CFA-induced rats

**Fig. 6:**
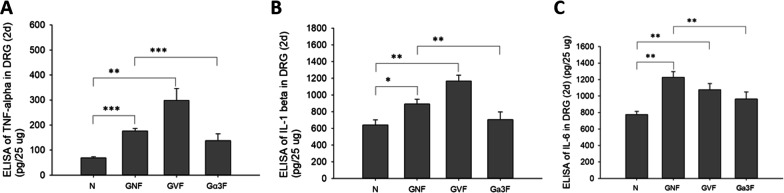
Effects of AAV-GlyRα3 on the expression level of cytokines in the L5 DRGs of CFA‑treated rats. For this experiment, CFA (100 μg/100 μl) hind paw injection was administered 8 weeks after the intrathecal injection of AAV-GlyRα3 (2.5 × 10^12^ vg). After 2 days of CFA treatment, L5 DRGs (25 μg/100 μl) were collected to evaluate cytokines expression. By ELISAs, the expression of **A** TNF-α, **B** IL-1β and **C** IL-6 was determined. The results are expressed as the means ± SE, and the data shown represent from three to five independent experiments. Mann–Whitney U test was used. *p < 0.05, **p < 0.01, ***p < 0.001

In vitro, we investigated the effects of pAAV, pAAV-GlyRα1, and pAAV-GlyRα3 on CFA-induced cytokine expression in F11 cells. The time schedule for the ELISA experiment is shown in Additional file 5: Fig. S5A. We found that tumor necrosis factor (TNF)-α (Additional file 5: Fig. S5B), IL-1ß (Additional file 5: Fig. S5C), and Il-6 (Additional file 5: Fig. S5D) were not induced in CFA-treated F11 cells or those transfected with pAAV, pAAV-GlyRα1, or pAAV-GlyRα3. In an in vivo experiment, we studied the potential involvement of AAV-GlyRα3 in CFA-induced neuron inflammation. The results showed that AAV-GlyRα3 substantially suppressed CFA-induced TNF-α (Fig. 6A), IL-1β (Fig. 6B) and IL-6 (Fig. 6C) activation in the DRG.

## Discussion

In our in vitro experiments, pAAV/pAAV-GlyRα1/3 transfection did not induce significant neuron cytotoxicity, ERK phosphorylation, or ATF-3 activation. In addition, PGE2-induced ERK phosphorylation was repressed in F11 cells by the expression of pAAV-GlyRα3. For pAAV/pAAV-GlyRα1/3 transfection efficiency, we used Lipofectamine in our transfection experiments. The transfection reagent Lipofectamine 2000 can induce cell damage (Zhong et al. 2008) and significantly decrease cell viability following pAAV or pAAV-GlyRα1/3 transfection.

In our current in vivo rat experiments, intrathecal injection of AAV or AAV-GlyRα3 successfully expressed GlyRα3 in DRG neurons and did not cause pain behavior. The histopathologic results showed that intrathecal NaCl injection followed by CFA intraplantar injection (GNF group) could cause more neurotoxicity than AAV plus CFA injection (GVF group) or AAV-GlyRα3 plus CFA injection (Gα3F group). However, the ATF-3 expression in DRG neurons was slightly different from our histopathologic results. This result suggested that following AAV, AAV-GlyRα3 intrathecally administered, and although neuronal cells remained intact (except when NaCl was intrathecally administered), slight neuronal injury was still induced as indicated by the increase in the expression of ATF-3, a marker of neuronal injury. The rat behavior did not significantly differ after intrathecal injection between the GNF, GVF, and Gα3F groups before CFA administration. However, the Gα3F group showed a significant analgesic effect compared with the GNF and GVF groups after CFA intraplantar injection. Additionally, when we observed the injected footpad, the rats in the Gα3F group had less focal footpad edema (pictures not shown). Together with the abovementioned results, AAV-GlyRα3 intrathecal injection in rats is safe and may be a potential tool for alleviating inflammatory pain.

Exogenous PGE2 was shown to directly induce ERK (but not p38) phosphorylation in DRG neurons (St-Jacques and Ma 2011) and other non-neuron types (St-Jacques and Ma 2011; Chen et al. 2007). Zhao et al. (2007) found a PGE2-dependent, ERK-regulated microglia-neuron signaling pathway that mediated the microglial component of pain maintenance. Our in vitro results indicated that exogenous pAAV-GlyRα3 administration can suppress PGE2-induced ERK phosphorylation in F11 neuron cells. Similar to the in vitro experiment, AAV-GlyRα3 intrathecal injection repressed ERK but not p38 phosphorylation in rats induced by CFA. The GlyRα3 subtype is associated with inflammatory hyperalgesia (Lynch 2004; Harvey et al. 2004b; Hosl et al. 2006). PKC-dependent phosphorylation of p38 and ERK has been reported (Kyriakis and Avruch 2012). The phosphorylation of ERK occurs in a PKA-independent manner (Laroche-Joubert et al. 2002). In contrast, Chen et al. showed that PKA stimulated p38 and ERK phosphorylation in breast adipose fibroblasts (Chen et al. 2007). Furthermore, a previous study found that p-ERK increased significantly in DRG neurons 8 h after PGE2 exposure; cotreatment of PGE2 with inhibitors of pan-PKA, pan-PKC, and ERK/mitogen-activated protein kinase (MAPK) significantly suppressed PGE2-induced IL-6 expression (St-Jacques and Ma 2011). In the present study (Wang et al. 2018), we found that p-ERK was expressed in DRG neurons of all sizes after CFA stimulation, but p38 was expressed only in small DRG neurons. In our previous study, we found that exogenous PGE2 induced GlyRα3 expression mostly in large DRG neurons. These findings may explain why AAV-GlyRα3 can reduce ERK phosphorylation by CFA but has no effect on p38 suppression.

Our study showed that pAAV-GlyRα3 transfection or administration of a glycine-receptor antagonist (strychnine) can downregulate PGE2-induced ERK phosphorylation in F11 neuron cells. There are two possible explanations for this. First, ERK phosphorylation is also controlled by GlyRs through the PKA/PKC-dependent pathway (Harvey et al. 2004b; Chen et al. 2007; Breitinger et al. 2018). Second, the glycine-receptor structure changes during strychnine binding, as does the internal domain binding site of PGE2-dependent PKA/PKC (Han et al. 2013; Huang et al. 2015). A previous study showed that the GlyRa3 architecture changed after strychnine binding. For an agonist or antagonist to bind with and affect the state of the channel, the signal must be transduced across the extracellular domain and transmembrane domain interface (Huang et al. 2015). In previous in vivo studies, mice with deficient or knocked out GlyRα3 not only lack inhibition of glycinergic neurotransmission by PGE2 but also show a reduction in pain sensitization induced by spinal PGE2 injection or peripheral inflammation (Harvey et al. 2004a; Xiong et al. 2012). Xiong also reported that nonpsychoactive cannabinoids can potentiate GlyRs, which significantly suppress chronic inflammatory and neuropathic pain (Xiong et al. 2012). According to these results, GlyRα3 deficiency or potentiation are involved in the PGE2-dependent inflammatory signaling pathway. Further studies are needed to elucidate the GlyR signaling pathways and identify additional potential molecular targets for inflammatory pain inhibition.

The proinflammatory cytokines TNF-α (Wei et al. 2007) and IL-6 (St-Jacques and Ma 2011; Lee et al. 2009) may be upregulated in DRG neurons after peripheral nerve injury. A previous study used a partial sciatic nerve ligation model to test IL-6 in DRG neurons and found upregulation of IL-6 in DRG neurons following nerve injury (St-Jacques and Ma 2011). Furthermore, increased IL-6 expression shifted from small- to medium- and large-sized damaged DRG neurons. Nerve injury models have also induced neuronal cell death, thereby inducing more proinflammatory cytokines, such as IL-6 (St-Jacques and Ma 2011; Zhao et al. 2007). CFA (Fang et al. 2013; Basting et al. 2019) are strong inflammatory mediators and can induce PGE2 synthesis in animal models. Our results showed that the expression of TNF-α, IL-1β, and IL-6 did not change in F11 cells treated with CFA or transfected with pAAV, pAAV-GlyRα1, or pAAV-GlyRα3.

A previous ex vivo study treated cultured sensory ganglion explants with a stabilized, long-acting PGE2 analog (dmPGE2) and showed that after high-dose dmPGE2 (100 μM) treatment, IL-6 expression increased significantly at 20 and 24 h (St-Jacques and Ma 2011). However, sensory ganglion explants were used, which differ from the F11 neuron line chosen for the present study. In addition to neurons, sensory ganglion explants include satellite glial cells, which are surround and can modulate neuron function. Satellite glial cells are also important immune regulators and can produce inflammatory mediators, such as prostaglandins, IL‐6, and TNF‐α. In addition, it has been suggested that cytokines in the nervous system are secreted by peripheral immune cells, microglia, astrocytes, and neurons. Our in vivo results showed that CFA intraplantar injection can induce TNF-α, IL-1β, and IL-6 expression in the DRG and that AAV-GlyRα3 can substantially suppress these proinflammatory cytokines.

Although our study showed that PGE2-induced ERK phosphorylation can be modulated by GlyRα3, and intrathecal AAV-GlyRα3 administration to SD rats suppressed CFA-induced ERK phosphorylation, the limitation of this study is that the mechanism of how GlyRa3 affects ERK phosphorylation is unclear, which needs to be clarified in future studies.

## Conclusions

The present study is the first to use AAV as a glycine receptor (pAAV-GlyR) vector to infect neuronal cells. PGE2 induced ERK phosphorylation in F11 neurons. Antagonists of the prostaglandin EP2 receptor, PKC, and glycine receptor can inhibit PGE2-induced ERK phosphorylation. We found that pAAV/pAAV-GlyRα1/3 transfection does not induce neuronal cytotoxicity, ERK phosphorylation, or ATF-3 activation in F11 neurons. Pretransfection with pAAV-GlyRα3 significantly suppressed PGE2-induced ERK phosphorylation. We suggest that PGE2-induced ERK phosphorylation can be modulated by GlyRα3. Furthermore, intrathecal AAV-GlyRα3 administration to SD rats significantly decreased CFA-induced inflammatory pain and suppressed CFA-induced ERK phosphorylation, did not significantly induce gross histopathological injury but elicited ATF-3 activation. More importantly, AAV-GlyRα3 significantly downregulated CFA-induced cytokine activation. These data suggest that intrathecal delivery of AAV-GlyRα3 is likely safe and may be a potential tool for alleviating inflammatory pain.

## Supplementary Information

**Additional file 1: Figure S1.** (A) pAAV-GlyRα1, (B) pAAV-GlyRα3 recombinant vector.

**Additional file 2: Figure S2.** Transfection efficiency and cell viability in response to pAAV, pAAV-GlyRα1, and pAAV-GlyRα3 transfection. (A) Time schedule for measuring the transfection efficiency of pAAV-GlyRα1 pAAV-GlyRα3. F11 cells were transfected with 2 µg or 5 µg of either (B) pAAV-GlyRα1 or (C) pAAV-GlyRα3 and GFP green fluorescence was measured 24, 48 and 72 h after transfection. (D) Time schedule for measuring cell viability by MTT assay F11 cells were transfected with 2 μg pAAV, pAAV-GlyRα1 or pAAV-GlyRα3 and incubated for 48 h. In addition, F11 cells were cultured for 48 h, serum free medium replaced the initial medium and was cultured for another 24 h, and then, PGE2 (100 μM) was added for 60 min in the end, F11 cells were harvested for MTT assay. (E) Relative cell viability is shown F11 cells grown in Lipofectamine free culture medium were used as a control. The white arrow indicates the time when the cells were collected to measure viability. The data are presented on the basis of at least three independent experiments. ***p < 0.001 vs control, one way ANOVA.

**Additional file 3: Figure S3.** Effects of PGE2 on p38 phosphorylation in F11 cells. (A) The time schedule of PGE2 administration and cell collection for measuring p38 phosphorylation. (B, C) The western blot results indicated that PGE2 did not increase the phosphorylation of p38 2 min, 5 min and 30 min after the administration of PGE2 (100 ng).

**Additional file 4: Figure S4.** Paw withdrawal response to a mechanical allodynia and thermal hyperalgesia were presented by comparison ipsilateral with contralateral side within (A) IT AAV GlyRα3 plus CFA injection group (Gα3F group), (B) IT AAV plus CFA injection group (GVF group), (C) IT NaCl plus CFA injection group (GNF group). Behaviour was assessed at day 0 (baseline); weekly for two months after intrathecal injection of AAV-GlyRα3 (2.5 × 10^12^ vg), AAV (2.5 × 10^12^ vg), or NaCl; and daily for four days after CFA (100 μl) injection. Mann Whitney U test, *p < 0.05, **p < 0.01, ***p < 0.001.

**Additional file 5: Figure S5.** Effects of pAAV, pAAV-GlyRα1, and pAAV-GlyRα3 on CFA-induced cytokine expression in F11 cells. (A) The time schedule for the ELISA experiment. (B) Tumor necrosis factor (TNF)-α, (C) IL-1β, and (D) Il-6 were not induced in CFA-treated F11 cells or those transfected with pAAV, pAAV-GlyRα1, or pAAV-GlyRα3.

## Data Availability

The datasets generated during and/or analyzed during the current study are available from the corresponding author on reasonable request.

## Abbreviations

TRPV1: Transient receptor protein vanilloid 1
NMDA: *N*-Methyl-d-aspartate
GABA: γ-Aminobutyric acid
GlyRs: Glycine receptors
DRG: Dorsal root ganglion
PGE2: Prostaglandin E2
PKA: Protein kinase A
PKCε: Protein kinase C epsilon
p-ERK: Phosphorylated extracellular signal-regulated kinase
IL-6: Interleukin 6
JNK: C-Jun N-terminal kinase
AAV: Adeno-associated virus
GFP: Green fluorescent protein
ATF-3: Activating transcription factor 3
SD rats: Sprague–Dawley rats
CFA: Complete Freund’s adjuvant
TNF-α: Tumor necrosis factor α
MAPK: Mitogen-activated protein kinase
