# Supplementary figures and images for: AAV-glycine receptor α3 alleviates CFA-induced inflammatory pain by downregulating ERK phosphorylation and proinflammatory cytokine expression in SD rats
Source: Mol Med. 2023 Feb 15;29:22. doi: 10.1186/s10020-023-00606-9 (PMC9933394; doi:10.1186/s10020-023-00606-9)

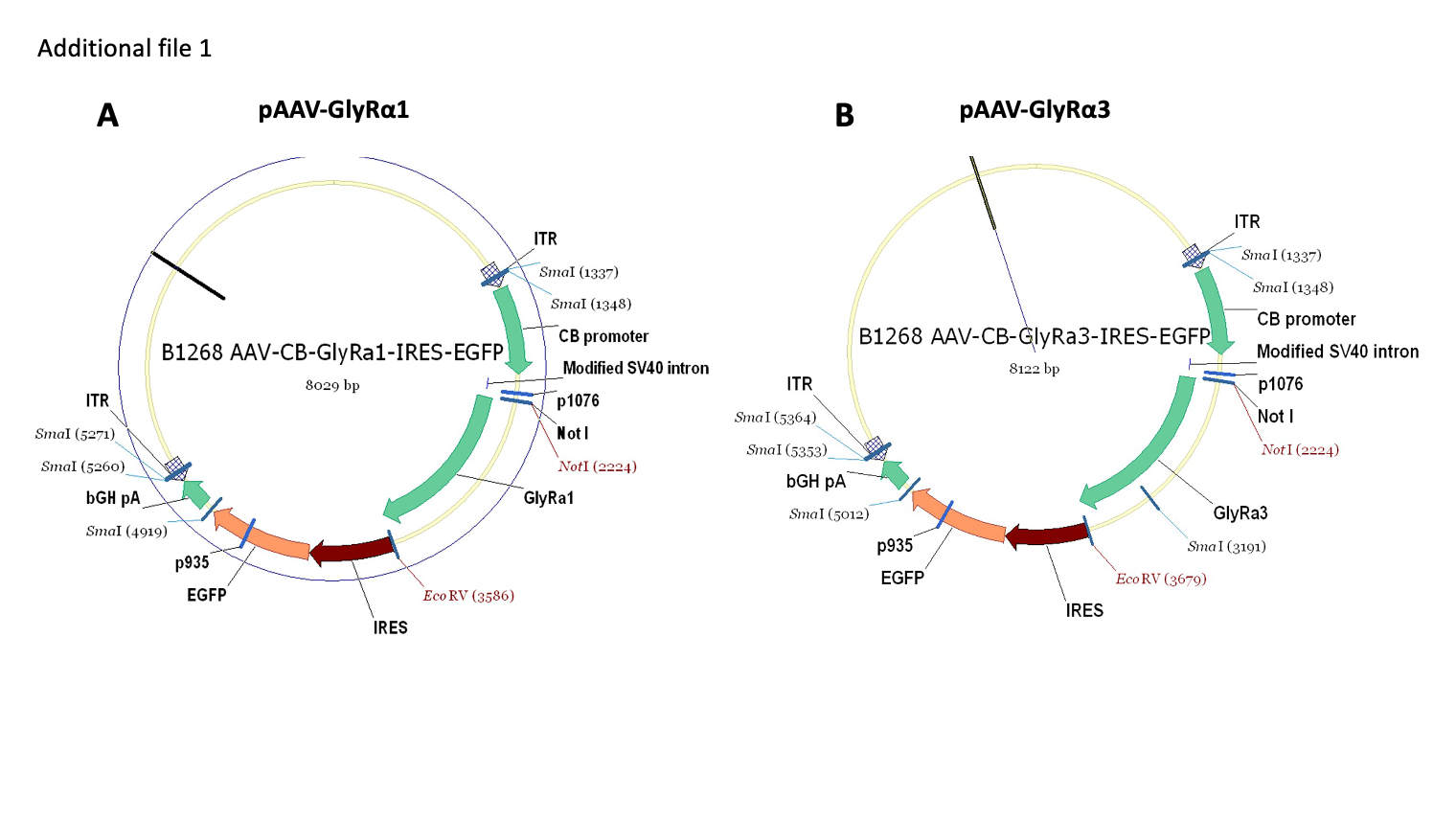

Supplement: Supplementary file 1 — Additional file 1: Figure S1. (A) pAAV-GlyRα1, (B) pAAV-GlyRα3 recombinant vector. [file 10020_2023_606_MOESM1_ESM.tiff]

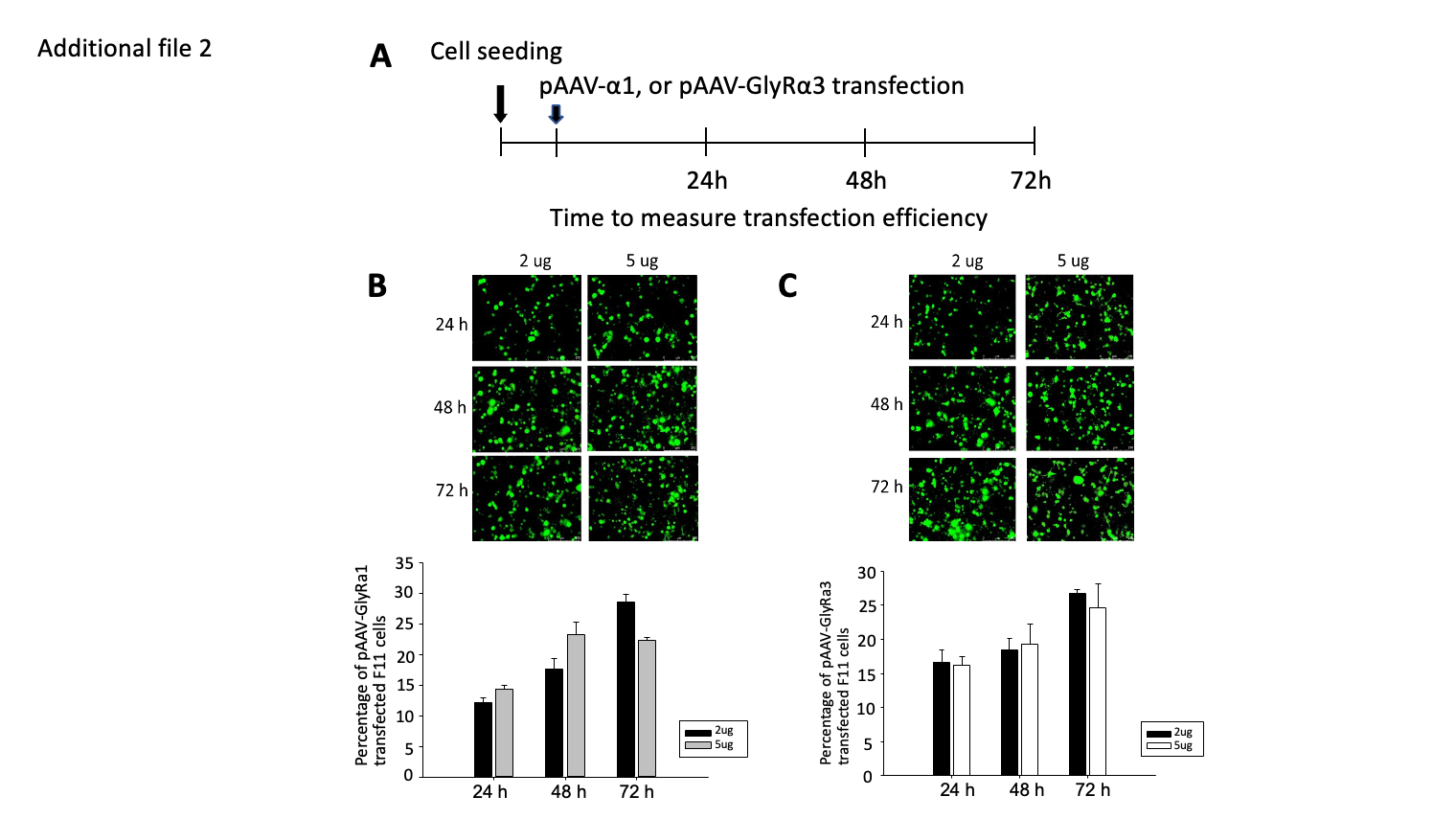

Supplement: Supplementary file 2 — Additional file 2: Figure S2. Transfection efficiency and cell viability in response to pAAV, pAAV-GlyRα1, and pAAV-GlyRα3 transfection. (A) Time schedule for measuring the transfection efficiency of pAAV-GlyRα1 pAAV-GlyRα3. F11 cells were transfected with 2 µg or 5 µg of either (B) pAAV-GlyRα1 or (C) pAAV-GlyRα3 and GFP green fluorescence was measured 24, 48 and 72 h after transfection. (D) Time schedule for measuring cell viability by MTT assay F11 cells were transfected with 2 μg pAAV, pAAV-GlyRα1 or pAAV-GlyRα3 and incubated for 48 h. In addition, F11 cells were cultured for 48 h, serum free medium replaced the initial medium and was cultured for another 24 h, and then, PGE2 (100 μM) was added for 60 min in the end, F11 cells were harvested for MTT assay. (E) Relative cell viability is shown F11 cells grown in Lipofectamine free culture medium were used as a control. The white arrow indicates the time when the cells were collected to measure viability. The data are presented on the basis of at least three independent experiments. ***p < 0.001 vs control, one way ANOVA. [file 10020_2023_606_MOESM2_ESM.zip › Additional file 2-1(R1)_ESM.tiff]

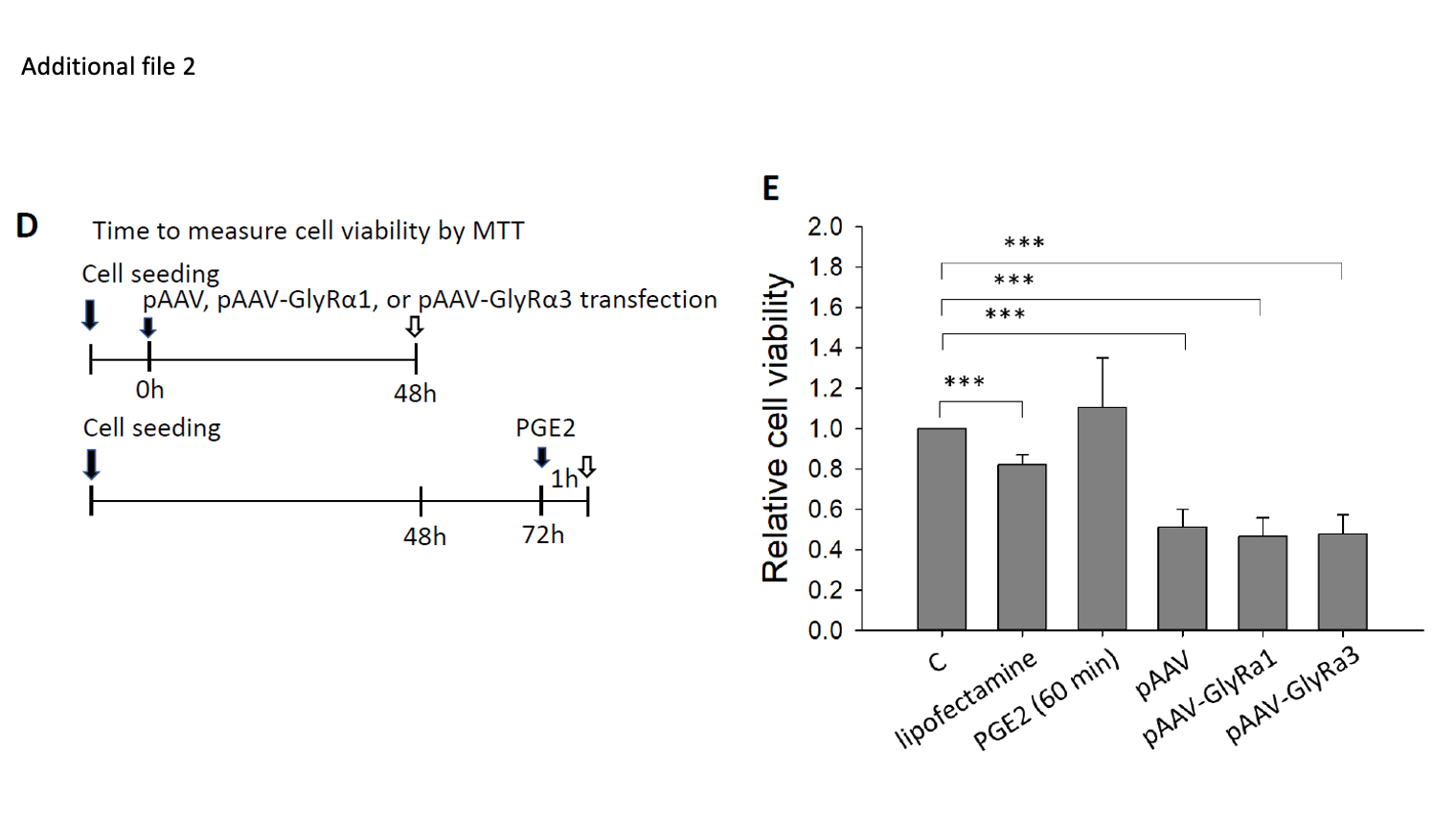

Supplement: Supplementary file 2 — Additional file 2: Figure S2. Transfection efficiency and cell viability in response to pAAV, pAAV-GlyRα1, and pAAV-GlyRα3 transfection. (A) Time schedule for measuring the transfection efficiency of pAAV-GlyRα1 pAAV-GlyRα3. F11 cells were transfected with 2 µg or 5 µg of either (B) pAAV-GlyRα1 or (C) pAAV-GlyRα3 and GFP green fluorescence was measured 24, 48 and 72 h after transfection. (D) Time schedule for measuring cell viability by MTT assay F11 cells were transfected with 2 μg pAAV, pAAV-GlyRα1 or pAAV-GlyRα3 and incubated for 48 h. In addition, F11 cells were cultured for 48 h, serum free medium replaced the initial medium and was cultured for another 24 h, and then, PGE2 (100 μM) was added for 60 min in the end, F11 cells were harvested for MTT assay. (E) Relative cell viability is shown F11 cells grown in Lipofectamine free culture medium were used as a control. The white arrow indicates the time when the cells were collected to measure viability. The data are presented on the basis of at least three independent experiments. ***p < 0.001 vs control, one way ANOVA. [file 10020_2023_606_MOESM2_ESM.zip › Additional file 2-2(R1)_ESM.tiff]

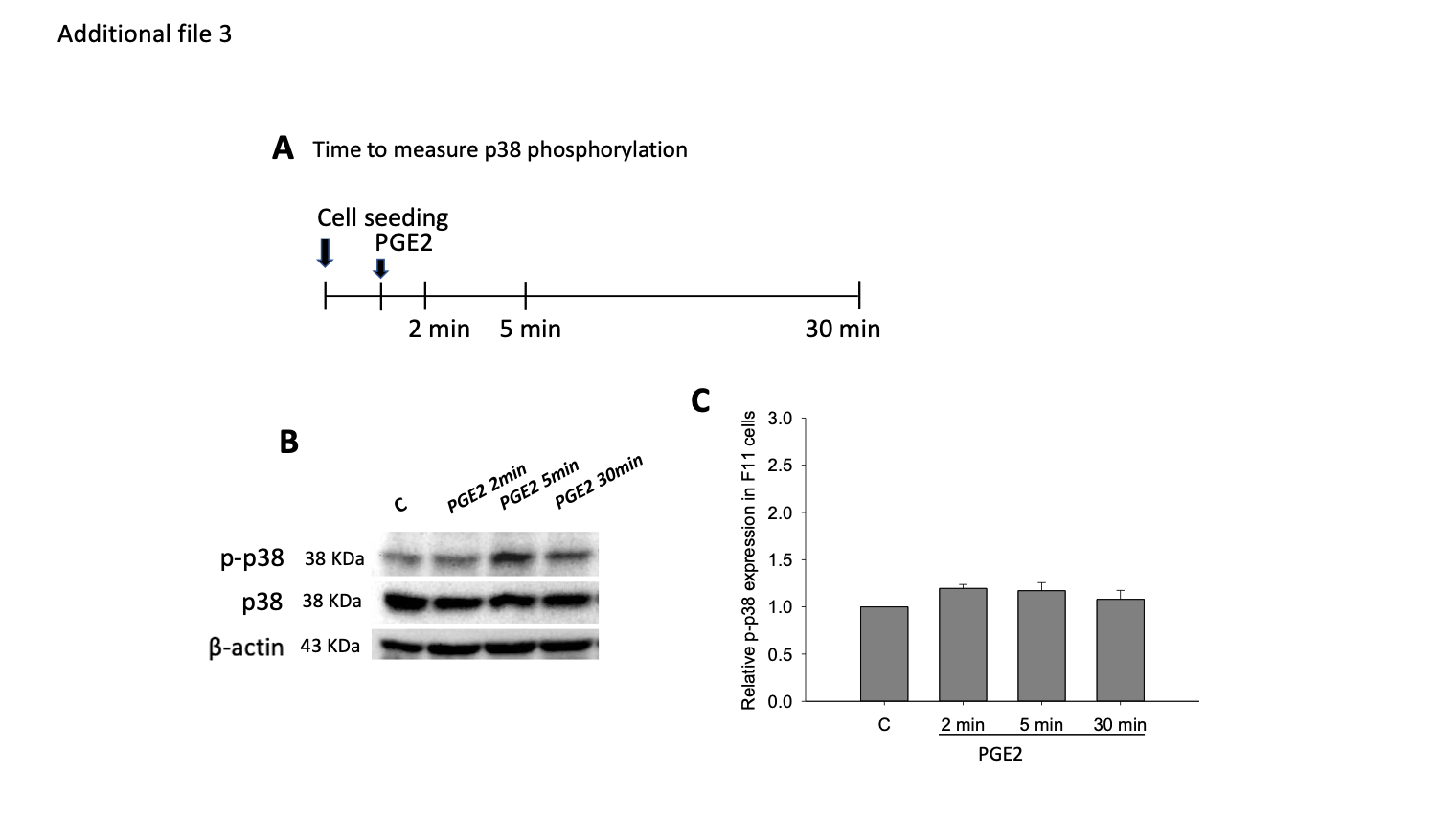

Supplement: Supplementary file 3 — Additional file 3: Figure S3. Effects of PGE2 on p38 phosphorylation in F11 cells. (A) The time schedule of PGE2 administration and cell collection for measuring p38 phosphorylation. (B, C) The western blot results indicated that PGE2 did not increase the phosphorylation of p38 2 min, 5 min and 30 min after the administration of PGE2 (100 ng). [file 10020_2023_606_MOESM3_ESM.tiff]

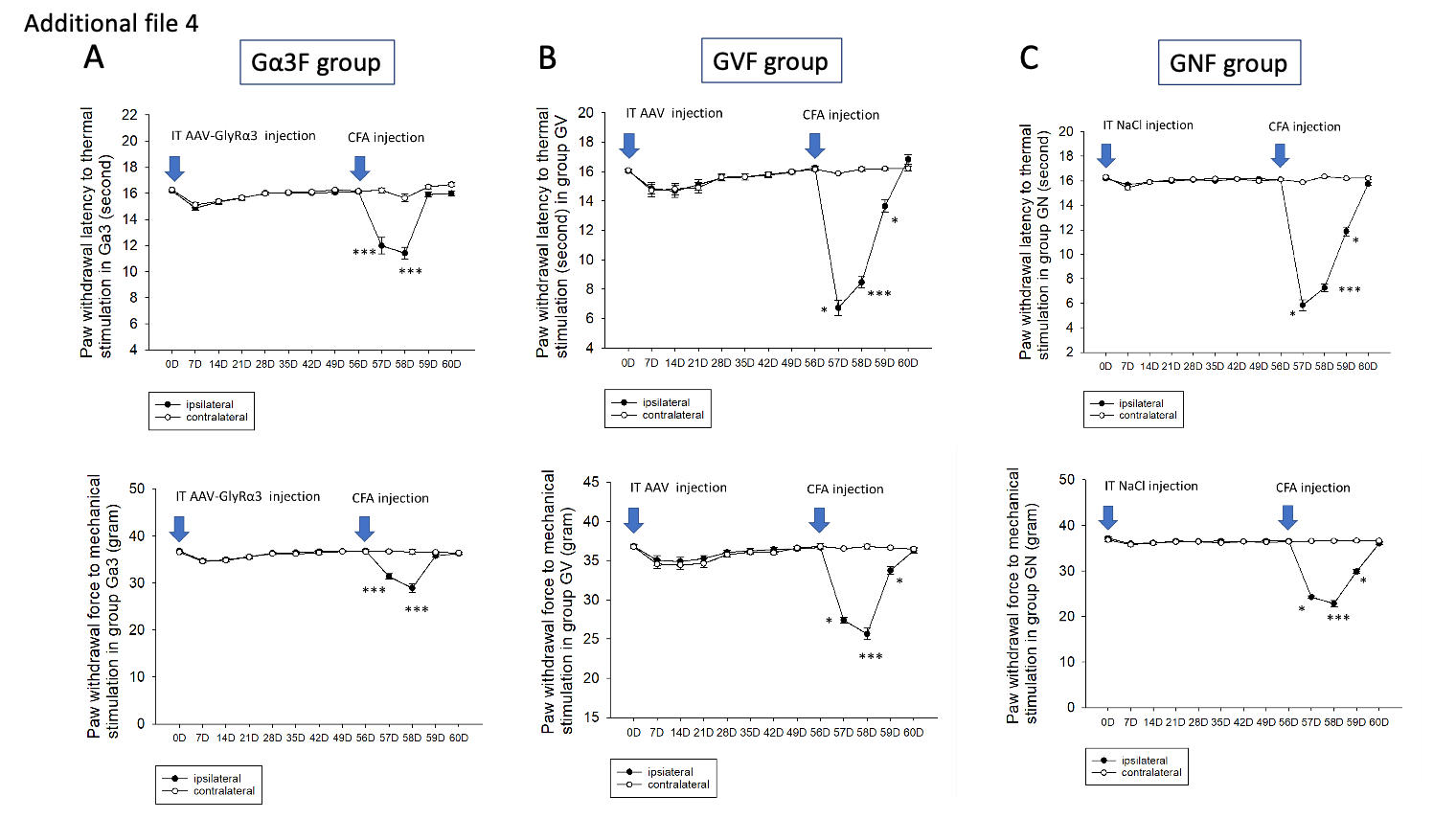

Supplement: Supplementary file 4 — Additional file 4: Figure S4. Paw withdrawal response to a mechanical allodynia and thermal hyperalgesia were presented by comparison ipsilateral with contralateral side within (A) IT AAV GlyRα3 plus CFA injection group (Gα3F group), (B) IT AAV plus CFA injection group (GVF group), (C) IT NaCl plus CFA injection group (GNF group). Behaviour was assessed at day 0 (baseline); weekly for two months after intrathecal injection of AAV-GlyRα3 (2.5 × 10^12^ vg), AAV (2.5 × 10^12^ vg), or NaCl; and daily for four days after CFA (100 μl) injection. Mann Whitney U test, *p < 0.05, **p < 0.01, ***p < 0.001. [file 10020_2023_606_MOESM4_ESM.tiff]

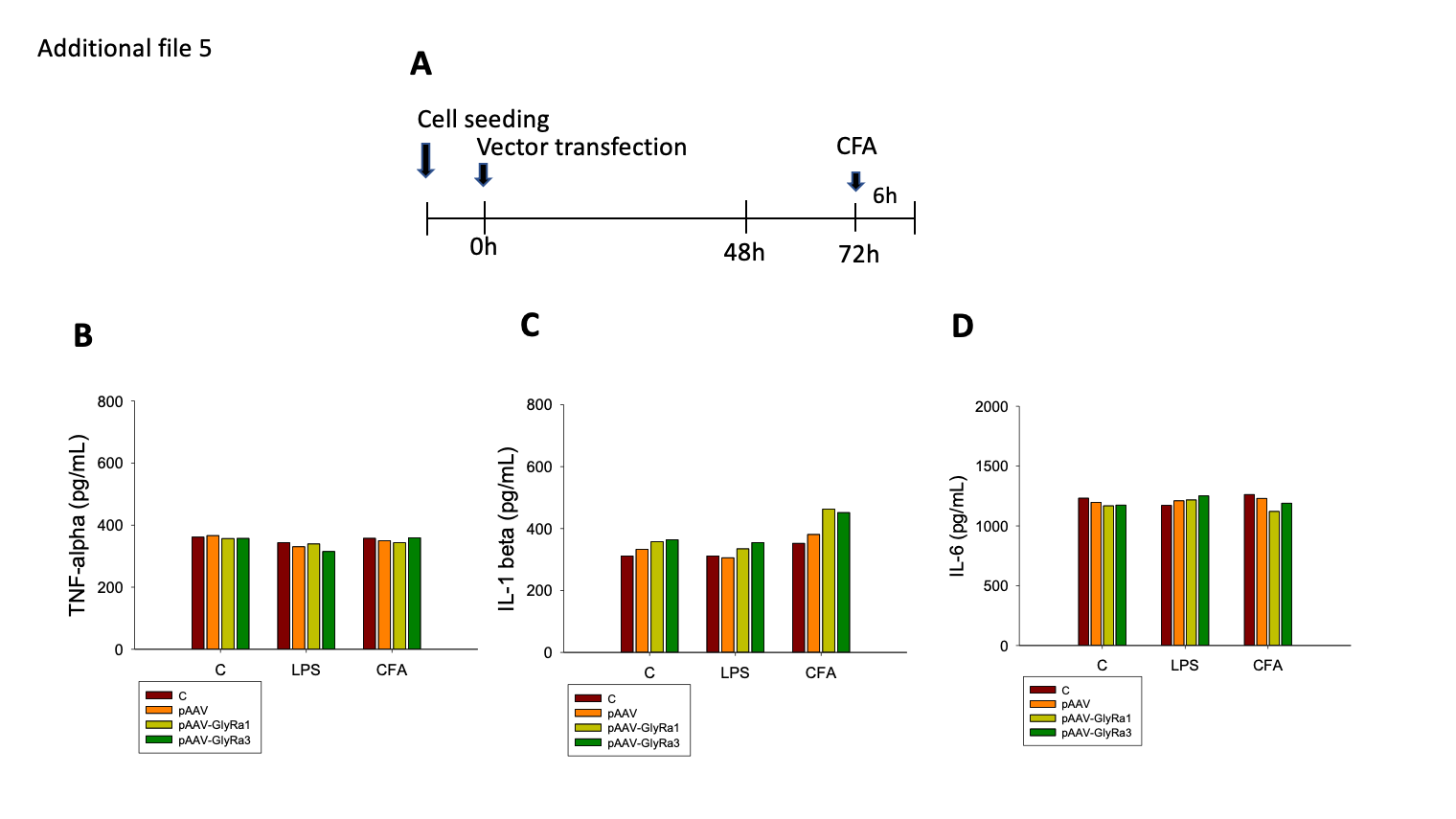

Supplement: Supplementary file 5 — Additional file 5: Figure S5. Effects of pAAV, pAAV-GlyRα1, and pAAV-GlyRα3 on CFA-induced cytokine expression in F11 cells. (A) The time schedule for the ELISA experiment. (B) Tumor necrosis factor (TNF)-α, (C) IL-1β, and (D) Il-6 were not induced in CFA-treated F11 cells or those transfected with pAAV, pAAV-GlyRα1, or pAAV-GlyRα3. [file 10020_2023_606_MOESM5_ESM.tiff]
